# Reframing Harm and Evidence: Advocacy Coalitions and the Transformation of Finnish Alcohol Policy

**DOI:** 10.1177/14550725261443300

**Published:** 2026-04-30

**Authors:** Josefin Westermarck, Anu Katainen, Matilda Hellman, Katariina Warpenius

**Affiliations:** 1 3835 University of Helsinki, Faculty of Social Sciences, Helsinki, Finland; 2 225313 University of Uppsala, Department of Sociology, Uppsala, Sweden; 3 3837 The National Institute of Health and Welfare, Alcohol, Drugs and Addiction Unit, Helsinki, Finland

**Keywords:** Alcohol, alcohol policy, stakeholders, public health, alcohol industry, advocacy coalitions, new public health

## Abstract

**Aims:**

The study examines how key stakeholders sought to influence the outcome of Finland's 2018 Alcohol Act reform in the formal consultation rounds.

**Methods:**

Drawing on the Advocacy Coalition Framework, we analyzed 224 stakeholder statements to uncover competing belief systems and framing strategies regarding alcohol-related harm.

**Results:**

We identified and named two main advocacy coalitions according to their standpoints on the reform's liberalization aspirations: the Public Health Coalition advocated for maintaining restrictive regulation based on population-level evidence and the Total Consumption Model, and the Alcohol Industry Coalition promoted deregulation through individual-level framings of responsibility and selective use of evidence. Both coalitions pursued epistemic dominance by reinforcing their own credibility and challenging that of their opponents. Although the Public Health Coalition relied on established scientific consensus, the Alcohol Industry Coalition appropriated the language of evidence-based policymaking to legitimize liberalization, aligning with broader neoliberal and populist discourses that question expert authority. Despite the scientific coherence of the public health side, the reform advanced in favour of deregulation, reflecting a broader shift toward a new public health and harm paradigm, in which individual responsibility is emphasized.

**Conclusions:**

The analysis shows how competing belief systems and discursive strategies shape alcohol policy in contexts where scientific expertise is increasingly politicized. It highlights a need for public health advocates to adjust their strategies to this new reality.

## Introduction

The alcohol policy system in Finland, like that of most Nordic countries, has traditionally been characterized by a restrictive approach based on a state alcohol retail monopoly. Its main aim has been to limit population-level alcohol consumption aiming to reduce alcohol-related harm, a broad term covering physical, social, and psychosocial consequences of alcohol use. Rooted in decades of epidemiological and social research, the Nordic policy model has emphasized the control of alcohol availability, price and marketing as the most effective way of reducing alcohol-related harm. A key theoretical foundation of this system has been the Total Consumption Model (TCM) (Bruun et al., 1975; Raninen & Livingston, 2020; Rossow 2019), which holds that changes in average alcohol consumption are systematically related to changes in the prevalence of heavy drinking and alcohol-related harm at the population level: when mean consumption increases, the entire distribution of drinking shifts upward, increasing the number of individuals exposed to high-risk levels of consumption and thereby raising the overall level of alcohol-related harm. Conversely, reductions in average consumption are associated with reductions in heavy drinking and related harms. Interventions for adjusting alcohol use have consequently targeted the population as a whole, not specifically high consumers. or problematic groups and contexts (Room & Livingston 2017).

In Finland a weakened centrality and influence of the TCM-based thinking has been witnessed especially . in two major policy reforms: In 2018 the sale of stronger alcoholic beverages was expanded from the alcohol monopoly to grocery stores andseveral restrictions were loosened affecting on-premise and off-premise sales (Karlsson et al., 2020). Another liberalization took place in 2024 when fermented beverages with up to 8% alcohol by volume were approved for sale in grocery stores, inflicting on the state alcohol monopoly's position (Warpenius & Katainen, 2026). These reforms stand out in a Nordic context, where other monopoly countries such as Sweden, Norway, and Iceland have largely demonstrated policy stasis (WHO 2025). Moreover, some European jurisdictions have decided to go in the opposite direction adopting more public health-oriented regulatory measures, as exemplified by Scotland's minimum unit pricing guidelines (Fergie, 2019) and Ireland's Public Health Alcohol Act (Lesch & McCambridge, 2021). Finland provides a unique contemporary natural experiment context for studying how evidence-based, population-oriented alcohol policy is renegotiated in a more neoliberal approach to individual consumer responsibility. This is a shift that is ascribed to the new public health era, in which risk and health are increasingly framed as matters of individual self-governance at the expence of more holisitic and structural whole-society-framings (Room, 2011).

The alcohol policy developments in Finland have occured in an increasingly polarized stakeholder environment, where different camps compete to define what counts as legitimate goals and evidence for alcohol policy-making. Stakeholders can be defined as actors or interest groups operating in a policy field, recognized as relevant parties with a vested interest in a policy domain (Sabatier & Weible, 2007). Previous research has identified two competing and particularly influential alcohol policy camps: alcohol industry and public health stakeholders (Katikireddi, 2014). Broadly speaking, public health-based alcohol policies are typically advocated by academic and medical institutions, health services, and health-related governmental and non-governmental organizations (Anderson & Baumberg, 2006; Thom, 2016). In contrast, alcohol industry stakeholders typically favour minimal market regulation and include producers and sellers of alcoholic beverages, trade and labour unions, financial think tanks, and consumer interest groups (Katikireddi, 2014).

Lesch and McCambridge (2021) characterize the alcohol policy arena as a politically charged epistemic battlefield where opposing coalitions frame evidence and concepts such as “harm” and “responsibility” in competing way. Public health stakeholders typically frame alcohol-related harm as a structural issue affecting entire societies and populations, whereas alcohol industry stakeholders emphasize evidence that frames health and lifetsyle choices in terms of individual responsibility (Severi & Hawkins, 2024). Alcohol policy stakeholder advocacy has therefore to some degree come to revolve around the strategic use and presentation of evidence, sometimes selectively interpreted or cherry-picked to support particular policy objectives (McCambridge et al., 2013).

In this study, we examine how alcohol policy stakeholder coalitions’ attempted to influence the 2018 Alcohol Act reform in Finland during the formal consultation phases. The reform concerned a set of changes including allowing grocery stores, kiosks, and gas stations to sell alcoholic beverages containing up to 5.5% alcohol by volume (previously limited to 4.7%) and opening up for drink mixtures diluted from strong alcoholic beverages (e.g., ready to drink alco-pops) to be sold in grocery stores. In addition it would permit licensed on-premise sale locations, such as bars and restaurants, to sell alcoholic beverages for off-premise consumption, and extended restaurant serving hours from 3 a.m. to 4 a.m. (Karlsson et al., 2020). Specifically, we examine how stakeholders involved in the consultations framed alcohol-related harm, in terms of for example individual versus population-level harm and state versus individual responsibility for preventing harm. These are studied as epistemic strategies for justifying either more liberal alcohol policies or the maintenance of restrictive measures. By unpacking these framings, we aim to further knowledge on how the epistemic and ideological foundations of Finnish alcohol policy are being renegotiated in a new alcohol policy landscape.

### Alcohol Policy Stakeholders as Advocacy Coalitions

During the parliamentary proceedings of the 2018 reform, opponents of restrictive measures found new momentum to challenge the traditional scientific consensus on alcohol regulation. Prime Minister Juha Sipilä's government (Centre Party, 2015–2019) had promised broad reforms of Finnish administration, working closely with the brewery-friendly National Coalition Party (Hellman 2012) and the populist Finns Party. By this point, pro-liberalization arguments that in previous times had been largely confined to right-wing politicians and to tabloid and business-oriented media had become mainstream and widespread on social media, increasingly framed by notions of individual freedom, anti-“nanny state” sentiments and post-truth critique of expert authority (Hellman & Karlsson, 2012; Hellman & Katainen, 2015; Ylä-Anttila, 2018).

It is against the backdrop of a steady byt recently intensified normalization of liberalization discourse that this study approach the epistemic reasoning underpinning the stakeholder comments. Our analysis applies the Advocacy Coalition Framework (ACF), which focuses on the role of stakeholder agency, belief systems, and coalition dynamics in shaping policy outcomes over time (Sabatier & Weible, 2007). The central premise is that policy subsystems — such as the one of alcohol policy — are populated by diverse actors, who share normative and causal beliefs and thus coordinate their activities to influence policymaking. The framework seeks to identify how coalitions form, how they are maintained, and under what societal and political conditions policy changes take place (Weible & Sabatier, 2018). Critiqued for underplaying the institutional and discursive contexts that enable or constrain their expression and influence (Schmidt, 2008) ACF still remains particularly suitable to understand “wicked” policy problems such as alcohol policy, characterized by disputes that are highly value-laden and ideologically polarized (Knai et al., 2021; Hiilamo, 2023).

Advocacy coalitions refer to clusters of stakeholders who express similar beliefs about the nature of policy problems and their ideal solutions, coordinating their strategies to influence policy processes. These coalitions are not temporary alliances but established parties of actors operating within a specific policy subsystem. ACF assumes that actors are guided by belief systems that structure their problem definitions, preferred policy outcomes, and definitions of legitimate evidence. First, *deep core beliefs* represent fundamental, ontological views about the world, grounded in general, hierarchical, social or normative values, and are relatively fixed and resistant to change. Second, *policy core beliefs* pertain to specific policy issues and define how these issues are framed, including preferences for specific policy outcomes. Lastly, *secondary beliefs* involve more detailed, technical or scientific information relevant to policymaking, such as evidence of causal relationships, the effectiveness of certain policy measures or interpretation of empirical data. Secondary beliefs typically surface in political debates about the practical means of achieving policy goals. They encompass scientific and technical explanations and can be selectively emphasized or minimized in coalitions’ broader belief systems (Weible & Sabatier, 2018), a key concern in alcohol policy debates.

Previous studies on alcohol policy stakeholders have mostly been conducted in Anglo-liberal contexts such as the UK and Australia, reflecting political systems commonly conceptualized as arenas of competition between organized interests (Hawkins & Holden, 2013; McCambridge et al. 2013; Savell et al., 2016). Research within this line of inquiry has shown that alcohol industry stakeholders often coordinate through coalitions to influence policy decisions, frequently presenting a unified position on key issues, even though internal divisions and competing interests may exist (Holden et al., 2012). Industry actors have been shown to influence alcohol policy through a combination of strategies, including direct lobbying of policymakers, coordination through trade associations and alliances, active engagement in consultation processes, and public communications that shape how policy problems are understood (Hawkins & Holden, 2013; Hawkins et al., 2012; McCambridge et al., 2018). Comparable strategies have been widely documented across other health-harming industries. Research on the Commercial Determinants of Health (CDH) shows that corporate actors in sectors such as tobacco, ultra-processed food, and gambling use similar political and discursive practices to shape policy and resist regulation (Ulucanlar et al., 2023). These parallels indicate that the ways in which alcohol industry operates to achieve preferred policy outcomes forms part of a broader pattern of corporate influence on public health policymaking (Kickbush et al., 2016).

A central element of these strategies concerns the selective mobilization of research and competing of what counts as policy-relevant evidence (Petticrew et al., 2018). Furthermore, the alcohol industry has a well-documented track record of funding and promoting research aligned with commercial interests (Jernigan, 2012). Through selective engagement with research, the alcohol industry typically highlights the efficiency of industry self-regulation, individual responsibility, and educational measures, although the effectiveness of these measures has proven weak in independent scientific evidence (Hawkins & Holden, 2014; Kypri et al., 2014). In this respect, industry actors not only use evidence, but also engage in practices of misrepresentation and creation of doubt by downplaying well-established links between alcohol and harms, or by portraying the evidence base as uncertain, overly complex, or insufficient (Vallance et al., 2020; Katainen et al., 2025).

Furthermore, a systematic review by Savell et al. (2016) identified five key argumentative strategies commonly employed by the alcohol industry: (1) alcohol consumption is normal and abstaining from alcohol is abnormal; (2) alcohol-related harm is limited to a small subgroup of problematic consumers; (3) alcohol marketing is harmless; (4) educating consumers is more effective than implementing strict public policies; and (5) the alcohol industry should be viewed as part of the solution rather than as contributing to the problem. Recent findings (Severi & Hawkins, 2024; Bujalski, 2025) show how such arguments form part of the alcohol industry coalition's belief system that legitimizes industry involvement in policymaking despite clear conflicts of interest.

Previous research from Anglo-liberal contexts has shown how the alcohol industry systematically positions itself as a legitimate policy partner while working to undermine the evidence base for effective population-level interventions such as minimum unit pricing (Hawkins et al., 2019). Such partnership initiatives are not merely rhetorical but serve as mechanisms for embedding corporate interests into policymaking in that they build long-term relationships with policymakers and occupying consultative roles in policy processes (Hawkins et al., 2012; McCambridge et al., 2018). An important mechanism by which this kind of partnership status is amplified is the use of language of corporate social responsibility (CSR), in which industry actors express their willingness to address alcohol-related harm (Mialon & McCambridge, 2018; Yoon & Lam, 2013). However, as Hellman (2012) points out, giving the rationalities promoted by different stakeholders equal weight can be problematic. For example, corporations have been shown to use CSR initiatives strategically to delay or influence policy processes and to utilize philanthropic sponsorships as brand marketing (Mialon & McCambridge, 2018).

These strategic dimensions of corporate influence remain rather under-examined in the Nordic alcohol policy scholarship which, in comparison to anglophone literature, has tended to adopt a more descriptive and institutionally oriented perspective on policy analyses. This difference reflects broader characteristics of Nordic political culture, which is characterized by state-centered corporatism, consensus-oriented and institutional trust. In comparison with the anglo-liberal contexts in which stakeholder analyses have been strong, Nordic countries have high structured cooperation between the state, labour-market organizations, and civil society, rather than open interest-group competition (Rothstein, 2001; Ihlen et al., 2021). In such systems, stakeholder participation is routinely institutionalized through corporatist consultation and negotiation procedures, embedding organized interests within formal policymaking structures. Consequently, Nordic alcohol policy research has traditionally focused on institutional arrangements, welfare-state development, regulatory traditions, and administrative governance logics (Tigerstedt, 2001; Karlsson, 2014), rather than on strategic competition and political behaviour of industry actors.

The ACF offers an interesting lens for studying current Finnish alcohol policymaking: while the system remains highly corporatist, it is simultaneously exhibiting increasing elements of more strategic alliance-building and confrontational tactics (Sama & Hiilamo 2019; Hellman & Katainen, 2015). For example, previous studies have observed the Finnish alcohol industry's increasingly bold use of partnership and responsibility discourses: In 2008 and 2012, the alcohol industry launched two CSR campaigns explicitly targeting alcohol-related harm, aiming to influence proposed restrictions on alcohol marketing (Sama & Hiilamo, 2019; Seuri et al., 2023; Hellman 2012). Stakeholders on the business side also engaged in direct lobbying of politicians in the Parliament and formed strategic alliances with grocery retailers (Sama & Hiilamo, 2019). Regarding the 2018 reform, Sama et al. (2021) show that prominent alcohol industry actors used Twitter to promote liberalization by emphasizing revenue generation, personal freedom, education, and individual responsibility as preferred responses to alcohol-related harm.

When it comes to public health advocates’ role and influence in alcohol policy it is a topic that has received less overall attention compared to studies on alcohol industry lobbying. In recent years, however, a growing body of international research has explored how public health actors engage with policy processes. In policy processes where alcohol policy has been tightened on public health grounds, alcohol-related harm has typically been framed as a question of population-level public health, such as in the policy debate surrounding minimum unit pricing in Scotland (Katikireddi et al., 2014) and the adoption of the Public Health (Alcohol) Act in Ireland (Lesch & McCambridge, 2021). Effective public health coalitions, such as those observed in the UK and Ireland, have strategically pooled limited resources and coordinated messaging to influence policy agendas (Lesch & McCambridge, 2021; Thom, 2016). Additional tactics have included forming formal networks between regional and national organizations (Schmitz, 2016) and agreeing upon long-term, persistent strategies communicated with a unified voice (Thom et al., 2016).

Several studies have identified challenges in alcohol policy initiatives grounded in a public health perspective. According to Košir (2024), efforts to influence alcohol policy from a public health perspective often struggle with limited resources and a lack of advocacy skills. Kypri et al. (2014), on their part, point to the selective and inconsistent use of evidence among public health actors. Moreover, the effectiveness of public health participation in formal policymaking remains uneven, as shown by mixed results from Scotland's licensing reforms (Fitzgerald et al., 2017). Overall, although recent work has expanded understanding of public health advocacy in alcohol policy, important gaps remain concerning how public health coalition articulate and justify their policy positions in direct interaction with policymakers, such as in lawmaking consultation rounds, and how public health belief systems are expressed and defended when confronted by industry framings of alcohol-related harm.

Our analysis focuses on stakeholder participation within the formal consultation rounds during the legislative process in 2017. Although advocacy coalitions employ numerous strategies to advance their policy objectives and much advocacy occurs prior to the legislative stage, formal stakeholder consultations remain a significant avenue to influence lawmakers (Katainen et al., 2025). Studying stakeholder arguments at this stage of the policy process highlights how contentious issues related to alcohol harm are navigated and debated in democratic processes that rely on expertise knowledge. Specifically, we examine which types of harm stakeholders consider relevant, how coalitions frame cause-and-effect relationships involving harm, and the justifications they provide for their positions.

## Methods

The data consist of all statements given in response to the government proposal (Government Bill 100/2017) regarding the new alcohol law. In Finland, the legislative process typically includes two rounds of consultation. In the first round, the ministry responsible for the law proposal invites written statements from parties that are considered relevant to the proposal. Although the ministry sends formal requests to selected stakeholders, the process is open to public participation allowing anyone to submit a statement. During the second round, statements are gathered in connection with hearings held by the parliamentary standing committees. During this stage, committees arrange consultations with experts and stakeholders they consider important in view of the proposed legislation.

In the first hearing round of the proposal, the Ministry of Social Affairs and Health received a total of 157 statements from government agencies (such as the Finnish Institute for Health and Welfare), ministries, municipalities, industry organizations, business associations, non-governmental organizations, and private citizens (27 statements). In the second round, the Social Affairs and Health Committee invited 67 experts to be heard. The Committee heard many of the same actors that had submitted statements during the first round, while also extending invitations to several university researchers. The total data from both rounds comprised 224 stakeholder statements. The longest statement came from the Finnish Federation of the Brewing and Soft Drinks Industry, consisting of 78 pages and 20,561 words, whereas the shortest statement from an individual citizen was 33 words long. Altogether, the documents add up to 835 pages of text, counted with an average of approximately 250 words per page.

The statements from both consultation rounds are publicly available online. Under current Finnish regulation, their study does not require ethical assessments or permissions. Still, due to the General Data Protection Regulation (i.e., GDPR), exposure of political opinions and the potential for unintentional incidental findings, ethical considerations were taken into account throughout the study (e.g., by removing names of persons that are mentioned in the material).

First, based on their stakeholder comments, the stakeholders were categorized into two coalitions according to their policy positions (Table 1). The coalitions were identified using the following criteria and eventually included 159 statements out of the total number of 224:

1. The organization demonstrated a clear material, institutional or ideological interest in the reform outcome, verified through its formal role or public communications on their official websites.
2. The stakeholder expressed an explicit policy stance in the statement either supporting or opposing increased alcohol availability as proposed in the bill.

**Table 1.** Policy Opinions and Stakeholder Groups Representing Alcohol Policy Coalitions.

	Public Health Coalition	Alcohol Industry Coalition
Policy stance	Opposing increased alcohol availability (105 stakeholders)	Promoting increased alcohol availability (54 stakeholders)
Stakeholder groups	Governmental organizations (i.e., GOs) and public authorities	Alcohol producers, especially the breweries, and their unions
	Non-governmental organizations (i.e., NGOs)	Alcohol retailers and on-premises sellers
	Academic researchers, universities, research institutes	Private research corporations, think tanks

Second, mentions of alcohol-related harm from both coalitions were counted and categorized into five subgroups (Table 2). Third, we classified the coalitions’ beliefs according to the three belief categories outlined in the ACF (Weible & Sabatier, 2018). Importantly, the coalitions identified in this study are analytical categories reconstructed from patterns of argumentation and shared policy beliefs, rather than formally constituted groups whose members necessarily self-identify as coalition participants. As discussed by Severi and Hawkins (2024), advocacy coalitions are rarely homogeneous or formally organized, and members may not necessarily perceive themselves as belonging to a common coalition. Similarly, although we identified two primary coalitions, this does not imply complete unanimity of views within each coalition. However, this approach allowed us to interpret stakeholder arguments not merely as isolated opinions but as expressions of broader belief systems consistent with the ACF logic.

1. **Deep core beliefs** were interpreted based on speakers’ general or normative positions toward Finland's alcohol policy. We asked what underlying values, worldviews or social imageries do the statements reflect?
2. **Policy core beliefs** were identified by examining each stakeholder's specific policy positions and justifications. We asked how the policy stances were connected to views concerning alcohol-related harm.
3. **Secondary beliefs** were identified by examining how stakeholders legitimized or justified their deep core and policy core beliefs (points 1 and 2) by referencing data or research evidence.

**Table 2.** Mentions of Different Alcohol-Related harms in Both Coalitions’ Statements (Number of Mentions).

	Public Health Coalition	Alcohol Industry Coalition
Harms to vulnerable groups	405	224
Societal harms	63	24
Health-related harms	68	15
Crime	42	1
Public safety	39	0

## Results

Both coalitions mentioned alcohol-related harms and recognized the harm as a problem. Table 2 shows variations of alcohol-related harms commented on and how harm-related comments were distributed between the coalitions. The following list of alcohol-related harm is ordered from the most to the least frequently mentioned in the data set: (1) harms to vulnerable groups; (2) societal harms; (3) health-related harms; (4) crime and (5) public safety. Moreover, Table 2 gives an idea of the kind of alcohol-related harms that were, overall, perceived as most relevant to address when appealing to the lawmakers. Additionally, Table 2 demonstrates the political practice of issue framing, specifically how alcohol-related harm was strategically emphasized by coalitions to influence policy outcomes. For example, both coalitions frequently highlighted harms concerning youth, families and children to persuade lawmakers to adopt their perspectives or policy objectives. Importantly, addressing alcohol-related harm was not solely stressed by representatives of the PHC; the AIC also grounded its policy objectives on the harm-based perspectives, but asserted individual-level strategies to mitigate such harms.

### The PHC

The PHC articulated two closely linked policy objectives: protecting public health and reducing the strain on the healthcare system caused by alcohol-related harm. These objectives were grounded in a deep core belief that society bears a collective responsibility to safeguard population health and wellbeing, particularly that of vulnerable groups. Within this framework, alcohol was viewed holistically, as a societal risk requiring regulatory intervention.

The changes in the Alcohol Act reform affect the public health of Finns, increasing both alcohol-related harm and alcohol-related deaths, as well as inequalities in health and well-being. Some of the proposed changes in the Alcohol Act are in conflict with Finland's welfare policy, which aims to, for example, reduce the welfare differences of the population. (Regional State Administrative Agency for Southwestern Finland)

The proposed policy recommendations made in the statements varied from maintaining the stricter alcohol policy measures to implementing a four-year trial period for the new law, raising alcohol taxes or even further restricting alcohol availability. The latter suggestion can be interpreted as accommodating a long-term scenario of balancing control policies by smoothing the transition in overall public health costs. According to the core policy belief of the PHC, alcohol availability regulation was seen as an essential instrument in reducing alcohol-related harm. In line with a TCM-framing, the decline in total alcohol consumption was seen less as a shift in drinking patterns and more as evidence of the state retail monopoly's success in limiting alcohol availability.

These core policy beliefs were supported by secondary beliefs drawing on scientific evidence and international guidelines. The coalition drew upon evidence from multiple sources, including the government proposal itself, governmental and local health authorities, official health guidelines, and academic literature. Additionally, it referenced international health-related frameworks from authoritative bodies such as the World Health Organization, the United Nations, the European Union, and the Organisation for Economic Co-operation and Development reflecting a common normative evidence-based global public health intituional context (Gostin et al. 2015). The policy positions of PHC were embedded in a network or culture of norms and language pertaining to public health-related international evidence-based standardization (see also Finnemore & Sikkink 1998).

A central element of the coalition's reasoning was the emphasis on evidence concerning alcohol-related mortality, mental health outcomes and the well-being of different population groups, as well as public safety and crime rates. By highlighting these widely empirically proven negative outcomes, the PHC combined empirical generalization (patterns of action and consequences), seeking to persuade legislators that the proposed policy reform was likely to lead to significant, wide-ranging harm. The primary justification for maintaining the existing alcohol policy framework relied consistently on references to TCM and its supporting research, advocating specifically for continued restrictions and preservation of the state retail monopoly. This structured line of argumentation was present in all of the PHC's statements.

SPEK [The Finnish National Rescue Association] is concerned about the change […] This change facilitates the availability of stronger alcoholic beverages, as a result of which it can be assumed that various accidents and incidents will increase. (The Finnish National Rescue Association, SPEK)

Based on the impact assessment in the proposal, we emphasize that the probability of harmful consequences is very high. As stated in the proposal, because the total consumption of alcohol would increase, empirically and based on theoretical information, it is certain that the change would have a negative effect on the social welfare, well-being and health of the population. (The Christian Democratic Party's Parliamentary Group)

The coalition presented a remarkably unified epistemic front, with stakeholders consistently aligning their perspectives on longstanding scientific proof, endorsing evidence by fellow coalition members and referencing each other's assessments, as exemplified in the quotation below. Similarly, the PHC questioned the assessments provided by the AIC concerning the proposed legislative change, critiquing the validity and integrity of corporation-funded research, as well as their research methodologies and the conclusions drawn from them. This reflects a cohesive front in terms of secondary beliefs and reveals intra-coalition epistemic stakeholder alliances.

The Kidney and Liver Association opposes the proposed amendments to the Alcohol Act, especially regarding raising the maximum strength. […] It has been estimated (including by the Institute of Health and Welfare, A-Clinic Foundation and The Finnish Medical Association) that the proposed changes would increase total consumption of alcohol and alcohol harm. This is also evident in the proposal. […] According to research data, one of the most effective ways to reduce alcohol consumption is to limit the availability of alcoholic beverages. (The Kidney and Liver Association)

The PHC's statements were characterized by a causal logic grounded in population-level evidence and established scientific knowledge. Their reasoning reflected a Bayesian mode of inference, assuming that empirically demonstrated causal relationships are likely to persist in future. This orientation positioned the PHC within what Yamaguchi (2025) terms a backward-looking restorative mode that seeks to uphold earlier institutional logics, bureaucratic practices, and welfare state principles. In this sense, PHC's argumentation can be seen as a defence of epistemic continuity and an attempt to preserve the authority of evidence-based policy in an increasingly uncertain and politicized environment.

### The AIC

The deep core belief of the AIC centred around the ordering of society around economic values, viewing alcohol policy primarily as an issue of market regulation. The proposed policy changes were framed as beneficial not only to the economy and consumers, but also to the society at large and as means to address alcohol-related harm. To persuade lawmakers, the coalition advanced a line of argumentation that framed alcohol-related harm as a result of an outdated alcohol policy in Finland. According to this view, the current alcohol policy was failing in two key areas: It was ineffective in reducing alcohol-related harm and it imposed excessive regulation on the alcohol industry, thereby restricting economic opportunities and limiting revenue generation that could otherwise benefit society.

As a remedy to the outdated and poorly working current alcohol policy, the AIC proposed alternative policy measures that, in their view, would create a “win–win” scenario, expanding market opportunities while simultaneously reducing harm. These proposed solutions focused on individual-level interventions, including behavioural “nudging” strategies to encourage moderate drinking, as well as information dissemination and health education. This represents a rhetorical inversion: while adopting the causal and evidence-based reasoning typical of the PHC, the AIC reinterpreted it to argue for the opposite policy direction, liberalization rather than control.

Alcohol-related harm was largely framed as an unfortunate but instrumental concern employed by public health actors to legitimize more stringent alcohol control measures. To advocate for deregulation, the industry not only acknowledged the issue of alcohol-related harm, but also proposed alternative solutions. For example, the AIC promoted the so-called “10% problematics” argument, suggesting that 10% of the population is responsible for virtually all alcohol-related harm, thereby shifting focus away from population-wide risks to downplay the broader public health impact.

To support their advocacy for increased alcohol availability, the alcohol industry coalition articulated its own beliefs about the relationship between availability and alcohol-related harm. An analysis of their secondary beliefs revealed that the coalition cited both academic research and studies conducted by independent research organizations — some of those referred to as independent were funded by the industry itself. This body of evidence was interpreted from two distinct angles. First, the coalition strategically utilized positive trends in declining alcohol consumption over recent decades in Finland to justify their policy stance. These developments were framed as evidence that existing concerns about alcohol-related harm were overstated or outdated. For instance, as the quote below illustrates, the decline of youth drinking was used to support a narrative that favoured liberalizing alcohol policy:

As far as young people are concerned, a positive reduction in alcohol use and binge drinking can already be witnessed. (The K-Retailers Association)

Second, the coalition employed a questioning approach that encompassed two key sub-themes. Aligned with their policy objectives, members of the AIC challenged the findings of scientific studies by selectively interpreting or oversimplifying the data and questioned the credibility of data sources and the accuracy of statements made by the PHC. As demonstrated in the quote below, research produced by public health authorities was strategically reinterpreted to support liberal policies:

There is a positive development in alcohol consumption among young people. Young peoples' alcohol consumption has decreased throughout the 2000s. According to the Institute of Health and Welfare (THL), it is already a megatrend. (The Federation of the Brewing and Soft Drinks Industry)

The TCM was challenged by the AIC from several angles. The overall reliability of the TCM was questioned by dissolving the model's core elements of availability, sales, consumption, risky consumption, and harm, treating them as separate and unrelated phenomena rather than as part of an evidence-based model of causality. The model's relevance and applicability in contemporary Finland was also criticized. The model was portrayed as outdated, with its empirical foundations deemed no longer reflective of modern societal dynamics or current patterns of alcohol use. Moreover, the coalition attempted to invert the logic of the TCM, arguing that increased alcohol availability and sales could lead to improved public health outcomes, primarily through generating greater state revenue. This paradigm is in sync with contemporary Nordic social policy ideology which is often justified in terms of economic performance (growth, productivity, labor supply) (Pedersen 2011).

Interestingly, the competing stances within the AIC was used to advance their shared policy objectives. The quote below illustrates how a mixed strategy was employed, combining selective interpretation of data with intra-industry differentiation. In this example, a major producer of mild alcoholic beverages, primarily beer, addresses alcohol-related harm by citing research on alcohol-related mortality. Rather than acknowledging any role of mild beverages in contributing to harm, the stakeholder redirects the blame toward producers of distilled spirits. Consequently, they advocated for stricter regulation on the availability of strong alcoholic beverages, while simultaneously promoting more lenient policies for mild ones:

Regarding the disadvantages, we would like to refer, for example, to the study on alcohol-related deaths published in 2016 by University of Helsinki docents [names removed]. According to it, the arrival of strong beer in grocery stores would not cause a significant change in the number of alcohol-related deaths and that their occurrence in Finland is mostly connected to the consumption of spirits, i.e., all distilled alcoholic beverages, but only to a small extent to the consumption of beer or wine. (Sinebrychoff Brewery)

All of these techniques can be seen as serving and privileging the language and logic of neoliberalism, understood as a structural project that values efficiency, individual responsibility, and market freedom (see Vivian 2006). At the same time, they reflect a populist tendency to dismantle established scientific causalities in alcohol policy debates. Here, the critique of expert authority does not stem from a rejection of complexity (Meyer 2023), but from an attempt to blur the established causal narrative linking alcohol availability to population-level harm. By selectively emphasizing evidence about individual choice and moderation, this discourse detaches the public health argument from its collective policy relevance, reframing alcohol harm as a matter of individual behaviour rather than structural regulation.

## Discussion

This study has examined how key stakeholder groups in Finnish alcohol policy sought to influence the 2018 Alcohol Act reform by framing alcohol-related harm. The two competing advocacy coalitions pursued epistemic dominance via utilizing secondary belief systems. Credibility of the opposing coalition's evidence was undermined and the legitimacy of their own was reinforced. Grounded in secondary beliefs (i.e., regarding evidence-informed views about which policy measures would be effective), the PHC positioned itself as a protector of public health and well-being. To support their position and persuade lawmakers, the coalition employed three primary strategies. First, it presented a unified scientific evidence base, with particular emphasis on the TCM as a central building block. Second, by consistently referencing and reinforcing each other's interpretations of research, coalition members built a strong epistemic alliance internally. Third, they critically assessed the policy alternatives proposed by the alcohol industry coalition, specifically questioning the methodological rigor and validity of industry-commissioned studies. The coalition advocated for a continuation of strong state regulation in line with Nordic corporatiism and its view on the neutrality of the state and its independent monitoring of the population.

In contrast, the AIC employed four tactics. First, the coalition framed harm as a consequence of a failing and overly restrictive policy, presenting deregulation as a more effective solution. Second, it challenged the TCM from multiple angles, questioning its scientific validity, relevance, and applicability in contemporary Finnish society. Third, the coalition supported its claims by introducing alternative forms of evidence, including industry-funded studies, using these to counter public health narratives and promote a more favorable interpretation of alcohol-related risks. Fourth, as an alternative to population-level regulation, the coalition promoted policy measures focused on individual behavior. Through these tactics, the alcohol industry coalition sought to shift the policy discourse toward a market liberalization — paradigm that works in tandem with a structural welfare state thining thanks to a neoliberal view on competition heightening the welfare state prosperity and by doing so contributing to population health and welfare.

Despite its Nordic context, these findings are consistent with international research on the political strategies of the alcohol and other health-harming industries (Savell et al., 2016; Kickbush et al., 2016). Similar framing practices, questioning scientific evidence, emphasizing personal responsibility, and legitimizing industry engagement, have been documented across different policy contexts. Our analysis demonstrates how such discursive strategies have come to operate and find a strong base within the specific institutional setting of Finnish policymaking, particularly in the studied formal stakeholder consultations. Our results highlight that the techniques of confrontational and interest-driven alcohol policy advocacy have gained a strong foothold in contemporary Finnish alcohol-policy making. Furthermore, our analysis reveals how established industry arguments are translated into the language of expert advice and evidence submission rather than being advanced through direct promotion of preferred policy outcomes. In this sense, our findings highlighting a convergence of populist expert critique and liberal market ideology reveal a significant shift in Finnish alcohol policy.

Despite the PHC's consistent argumentation against the legislative change, it was unable to sufficiently persuade lawmakers and the Government's proposal was accepted. Our analysis suggests that the AIC successfully framed the proposed law as a responsible way of advancing business interests. Rather than sidestepping concerns about increased alcohol-related harms, the industry coalition directly engaged in these issues, responding in a manner that partly mimicked the public health coalition's rationale; namely, by strategically appealing to research evidence. This confirms earlier findings that industry actors increasingly rely on the language of evidence informed policymaking to enhance legitimacy (McCambridge et al., 2018; Katainen et al., 2025), reflecting what Hawkins and Ettelt (2019) describe as a rationalist understanding of policymaking in which disputes are expected to be settled by appealing to empirical evidence alone.

The PHC presented a cohesive, science-based case centred on population-level regulation, framing it as the sole evidence-based approach and emphasizing worst-case harm scenarios over potential trade-offs (Room, 2011). This narrowed the evidentiary scope through selective use of evidence, including limited engagement with the contextual limitations of the total consumption model. This tendency has also been noted in public health advocacy by Kypri et al. (2014), who caution against over-reliance on singular models without contextual nuance. Finally, by forming epistemic alliances and disregarding alternative policy proposals, the coalition contributed to a form of epistemic closure. This dynamic, where internal consensus is prioritized over critical engagement, can ultimately constrain the policy discourse and limit broader debate on potential regulatory approaches. By identifying this dynamic in a Nordic policy context, the present study extends previous analyses of stakeholder framing by illustrating how both public health and industry coalitions contribute to reinforcing epistemic boundaries in policy debates.

Within legislative processes, consultations function not only as sources of expert input, but also as instruments of political legitimation, enabling lawmakers to justify policy choices by reference to authoritative expertise (Grundmann & Stehr, 2012). Such consultations often reinforce pre-existing political positions because experts and stakeholders may be selectively engaged to support predetermined policy preferences (Kropp & Wagner, 2010). In the 2018 reform, coalition arguments thus served a dual purpose: providing evidence-based rationales for policy positions while allowing legislators to claim broad expert and stakeholder support. Operating in an already favourable policy environment, the AIC strategically addressed alcohol-related harms by reframing public health arguments through selective appeals to research evidence, whereas the PHC adopted a defensive stance, seeking to demonstrate that the reform would increase harm and public health costs, comprising concerns that were not central to the reform's underlying rationale.

A key strength of the present study is that it systematically examines the argumentative strategies of the main advocacy coalitions in the alcohol policy field in a formal legislative setting. By analysing how these coalitions operated in the parliamentary consultation process, the study offers new insight into the dynamics of alcohol policymaking in Finland, illustrating the gradual transformation of the traditional Nordic alcohol policy model. A limitation of the study is that the data do not allow for a broader assessment of the coalitions’ influence or the different ways in which they have sought to promote their policy positions.

## Conclusions

The present study accounts for sentiments and epistemic argumentation among stakeholder alliances in the key processes leading up to more liberalized alcohol control policy in contemporary Finland. It suggests a principle shift from a Nordic state-controlled alcohol policy paradigm to more individualized responsibility-centred alcohol policy frameworks aligned with a new public health approach (Room, 2011, The identified public health coalition′s evidence-based focus on population-level regulation may have unintentionally contributed to the diminishing influence of traditional public health advocacy. By presenting population-level restrictive alcohol policy as the only legitimate solution, the PHC may have distanced itself from necessary contact surfaces with notions of individual responsibility, consumer choice, and economic freedom. The shift in what is considered justifieable and legitimate underpinnings of alcohol policy implicates severe challenges for the future of public health-based alcohol policy in Finland. As political and cultural attitudes increasingly detach from state intervention, both traditional evidence-based public health advocacy and scientific authority are likely to loose grounds. To remain influential the PHC may need to reframe or repackage its core messages and strategies..
